# Reproductive biology and nectar secretion dynamics of *Penstemon gentianoides* (Plantaginaceae): a perennial herb with a mixed pollination system?

**DOI:** 10.7717/peerj.3636

**Published:** 2017-08-09

**Authors:** Lucía Salas-Arcos, Carlos Lara, Juan Francisco Ornelas

**Affiliations:** 1 Doctorado en Ciencias Biológicas, Universidad Autónoma de Tlaxcala, Tlaxala, Mexico; 2 Centro de Investigaciones en Ciencias Biológicas, Universidad Autónoma de Tlaxcala, Tlaxcala, Mexico; 3 Departamento de Biología Evolutiva, Instituto de Ecología AC, Xalapa, Veracruz, Mexico

**Keywords:** Hymenoptera, Plantaginaceae, *Penstemon*, Hummingbirds, Mixed-pollination syndrome

## Abstract

**Background:**

In many plant species, pollination syndromes predict the most effective pollinator. However, other floral visitors may also offer effective pollination services and promote mixed pollination systems. Several species of the species-rich *Penstemon* (Plantaginaceae) exhibit a suite of floral traits that suggest adaptation for pollination by both hymenopterans and hummingbirds. Transitions from the ancestral hymenopteran pollination syndrome to more derived hummingbird pollination syndrome may be promoted if the quantity or quality of visits by hummingbirds is increased and if the ancestral pollinator group performs less efficiently. The quantification of such shifts in pollination systems in the group is still limited. We aimed to investigate floral traits linked to this pollination syndrome in *Penstemon gentianoides* with flowers visited by bumblebees and hummingbirds.

**Methods:**

We investigated the floral biology, pollinator assemblages, breeding system and nectar production patterns of*****P. gentianoides* inhabiting a temperate montane forest in central Mexico. Pollination experiments were also conducted to assess the pollinator effectiveness of bumblebees and hummingbirds.

**Results:**

*P. gentianoides* flowers are protandrous, with 8-d male phase (staminate) flowers, followed by the ∼1–7 d female phase (pistillate phase). Flowers display traits associated with hymenopteran pollination, including purple flowers abruptly ampliate-ventricose to a broad throat with anthers and stigmas included, and long lifespans. However, the nectar available in the morning hours was abundant and dilute, traits linked to flowers with a hummingbird pollination syndrome. Two hummingbird species made most of the visits to flowers, *Selasphorus platycercus* (30.3% of all visits), followed by *Archilochus colubris* (11.3%). Bumblebees (*Bombus ephippiatus*, *B. huntii* and *B. weisi*) accounted for 51.8% of all recorded visits, but their foraging activity was restricted to the warmer hours. Hummingbirds made more foraging bouts and visited more flowers than hymenopteran species. Flowers experimentally pollinated by *B. ephippiatus* produced significantly more fruits than those pollinated by *S. platycercus*. However, there was no statistical difference in the number of seeds produced per fruit when a bumblebee or a hummingbird was the pollinator.

**Conclusions:**

We have shown that bumblebees and hummingbirds visit and pollinate *P. gentianoides* flowers. Despite floral traits resembling the hymenoptera pollination syndrome, flowers of *P. gentianoides* offer characteristic nectar rewards to flowers with a hummingbird pollination syndrome. Although pollination efficiency is higher among flowers visited by hymenoptera, the noteworthy percentage of fruit production and number of seeds per fruit derived from hummingbird pollination highlights the importance of hummingbirds as a functional group of pollinators that might have potential evolutionary consequences to the plants.

## Introduction

Animal pollination is the basis of pollen transfer and directed outcrossing, and the selection exerted on plant traits represents an important force in the process of reproductive isolation and speciation underlying the diversification of flowering plants (Ollerton, 1999; Thompson, 2001; Hu et al., 2008; Kay & Sargent, 2009; Toon, Cook & Crisp, 2014). Animal pollinators obtain food rewards from the flowers they visit, most commonly pollen or nectar, leading to asymmetric interactions with divergent ‘interests’ between plants and their pollinators (Proctor, Yeo & Lack, 1996). To maintain pollinator services, some flowering plants have evolved suites of floral traits and specialized mechanisms to filtering particular functional groups of pollinators to act as the main pollinators (Van der Pijl, 1961; Stebbins, 1970; Shivanna & Tandon, 2014). However, the association of particular sets of floral traits with specific pollinators (so called ‘pollination syndromes’; Faegri & Van der Pijl, 1979; Rosas-Guerrero et al., 2014) has been questioned given the widespread generalization in pollination systems (e.g., Waser et al., 1996).

Although insects represent the most important pollinators of flowering plants, pollination by vertebrate animals plays also a fundamental role (Proctor, Yeo & Lack, 1996). In the Americas, hummingbirds (Trochilidae) can support pollination of around 15% of Angiosperms in any environment (Buzato, Sazima & Sazima, 2000). These plants generally possess very distinctive floral traits (the pollination syndrome of ornithophily) from those pollinated by insects (Van der Pijl, 1961; Faegri & Van der Pijl, 1979). Flowers pollinated by hummingbirds are distinguished by presenting red and alike colors, dilute nectar, amino acids in low concentrations, and a high proportion of sucrose (Brown & Kodric-Brown, 1979; Freeman et al., 1984). Other morphological traits include anthers and stigma protruding from the corolla, a narrow and tilted floral tube, soft pedicels, and limited or absent landing structures; they are scentless and pollen is generally displayed on a single event (Martínez del Rio, 1990; Thomson et al., 2000; Castellanos, Wilson & Thomson, 2004; Thomson & Wilson, 2008). On the other hand, flowers pollinated by diverse type of bees (melittophily) usually show blue, violet, white or yellow colors and they present low quantities of nectar of a sticky, viscous consistency containing a low ratio of sucrose (Proctor, Yeo & Lack, 1996). They usually have landing platforms and nectar guides are frequent and elaborate (Dafni & Giurfa, 1999). Also, anthers and stigma usually stand within the corolla and in most cases, the floral tube is wide; they scent and the pollen is displayed gradually in several events (Thomson et al., 2000; Wilson et al., 2004; Wilson et al., 2007). However, many plant species pollinated by hummingbirds do not fit the ornithophilous pollination syndrome traits.

Native to North America, *Penstemon* (Plantaginaceae) species (280–284 perennial herbs and sub-shrubs) show a wide floral variety and pollination systems (Wolfe et al., 2006; Wilson et al., 2007; Wessinger et al., 2016). Although most species have flowers pollinated by diverse type of insects (bees, bumblebees, butterflies, wasps, flies), flowers of 41 species are hummingbird-adapted (Lyon & Chadek, 1971; Bateman, 1980; Clinebell & Bernhardt, 1998; Tepedino, Sipes & Griswold, 1999; Wilson et al., 2004; Tepedino et al., 2007; Wilson et al., 2007; Lara & Ornelas, 2008). Traits associated with hymenopteran pollination in this group of *Penstemon* species include blue or purple short corollas, petals joined as a landing platform, and reproductive structures included within such corollas (Thomson et al., 2000). In contrast, *Penstemon* species pollinated by hummingbirds usually display red long tubular corollas, exserted reproductive structures and landing platforms, but the petals are joined (Wilson et al., 2004). Within the genus, however, there are species that overlap between these pollination syndromes. When this occurs, plants show suites of floral traits linked to attract and reward both pollinator types and both insects and hummingbirds transfer and deposit pollen effectively (Reid, Sensiba & Freeman, 1988; Lange & Scott, 1999; Lara & Ornelas, 2008). These pollination systems have been considered as transitions between pollination syndromes, i.e., intermediate stages regarding shifts from the ancestral hymenopteran ‘bee’ pollination toward more derived hummingbird pollination (Wolfe et al., 2006; Wilson et al., 2006; Wilson et al., 2007; Lara & Ornelas, 2008; Thomson & Wilson, 2008; Wessinger, Hileman & Rausher, 2014; Wessinger et al., 2016). The existence of mixed pollination between vertebrates and insects has been demonstrated experimentally and also has been studied from the phylogenetic point of view in other plant genera such as *Inga* (Fabaceae; Amorim, Galetto & Sazima, 2013), *Faramea* (Rubiaceae; Maruyama, Amorim & Oliveira, 2010), *Encholirium* (Bromeliaceae; Queiroz et al., 2016), and *Drymonia* (Gesneriaceae; Ramírez-Aguirre, Martén-Rodríguez & Ornelas, 2016).

*Penstemon gentianoides* (Kunth) Poir. is a species with melittophilous floral traits, which suggest bumblebee pollination (Wilson et al., 2004). However, several hummingbird species were reported defending intensively the floral patches of this species (Lara, 2006). Here we investigated the pollination biology of *P. gentianoides* over a 3-year study and hypothesized a mixed pollination system. Specifically, we focused on the pollination biology and breeding system of *P. gentianoides*, and described its floral visitors, quantified nectar secretion dynamics, and determined the pollinator effectiveness of main floral visitors of a *P. gentianoides* population inhabiting a temperate montane forest in central Mexico. The study was centered on the following questions: (1) Are the suites of floral traits promoting the visits of both hymenopteran (ancestral pollination group) and hummingbirds (derived pollination group); (2) is the quantity or quality of visits increased when visited by hummingbirds; and (3) are bumblebees less effective pollinators than hummingbirds?

## Methods

### Field study permissions

We obtained permits for fieldwork from the Mexican government to conduct this work from the Secretaría de Medio Ambiente y Recursos Naturales, Instituto Nacional de Ecología, Dirección General de Vida Silvestre (permit number: INE, SEMARNAT, SGPA/DGGFS/02439/16/0296). The permit specifically allowed us for fieldwork at the study area though additional permits from municipal and community authorities of La Malinche National Park.

### Study sites

Fieldwork was carried out from July to November 2014 and from July to December 2015–2016 in a temperate montane forest located at the La Malinche National Park, in the state of Tlaxcala, Mexico (19°15.205′N, 098°02.080′W; at 3,700 m above sea level, m a.s.l.). Mean annual precipitation is 800 mm, the rainy season is between June and October, and mean annual temperature is 15 °C. Coniferous forest is the dominant vegetation type. Above 3,500 m a.s.l., pine forest contains pure stands of *Pinus hartwegii* Lindl. and *Abies religiosa* (Kunth) Schltdl. & Cham. The main shrubs are *Baccharis conferta* Kunth and *Eupatorium glabratrum* Kunth and herbaceous plants include *Senecio platanifolius* Benth., *Muhlenbergia macroura* (Kunth) Hitchc., and *P. gentianoides* (Lara, 2006; Villers, Rojas & Tenorio, 2006).

### Study species

**Figure 1:**
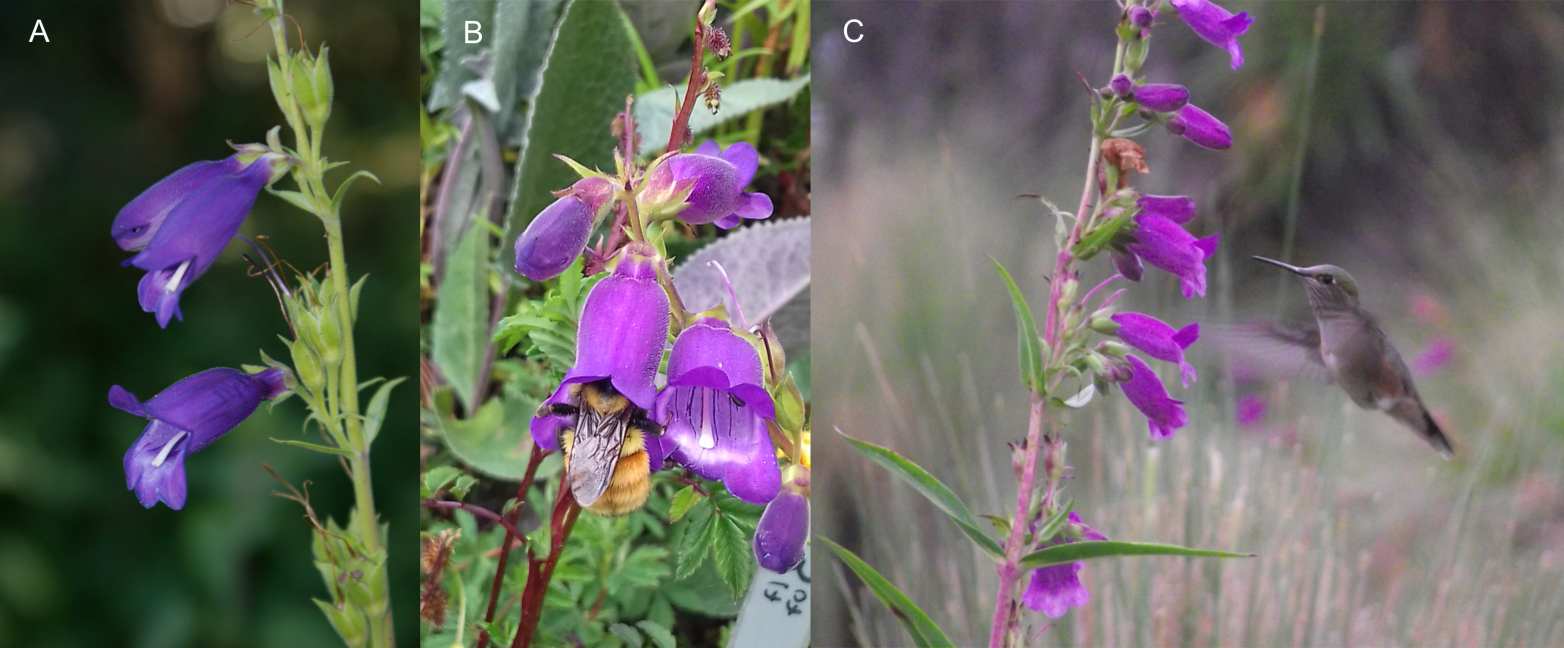
Morphology and floral visitors of *Penstemon gentianoides* (Kunth) Poir. at La Malinche National Park, Tlaxcala, Mexico. (A) Inflorescence and lateral view of *P. gentianoides* pendant flowers. Note the broad purple corolla tubes that allow large bumblebees to reach the nectaries, lower lips that extend as a landing platform, anthers and stigmas nearly included, and a stiff pedicel that holds the flowers nearly horizontal. (B) *Bombus ephippiatus* resting on a flower of *P. gentianoides*, while accessing nectar. (C) *Selasphorus platycercus* hovering and taking nectar during a visit to *P. gentianoides*. Photos by Carlos Lara, Lucía Salas and Magali Luna.

*Penstemon gentianoides* is an herbaceous perennial plant commonly found in pine and fir forests (3,000–4,200 m a.s.l.) along the Trans-Mexican Volcanic Belt (Michoacán, State of Mexico, Puebla, Tlaxcala, Oaxaca and Veracruz) southward into Chiapas and Guatemala (Straw, 1962). Individuals (0.5–1.5 m high) bear 15–25 paniculate inflorescences, each with 2–4 pendant flowers from terminal branching stems opening per day, and ∼90 floral buds may eventually reach the flower stage during the blooming season (4 months), which extends from July to November for the region (Lara, 2006). Traits that enable *Penstemon gentianoides* to exploit hymenopteran pollination include blue, violet or purple and vestibular flowers abruptly expanding into a broadly inflated throat and a prominent lower lip with anthers and stigmas nearly included (Calderón de Rzedowski & Rzedowski, 2002; Fig. 1). However, Lara (2006) observed large hummingbird species (*Colibri thalassinus*, *Eugenes fulgens*, *Lampornis clemenciae*) defending floral patches of *P. gentianoides* at lower elevation in the La Malinche National Park.

### Floral biology

During the blooming season (July–November 2014), we selected 125 plants and measured two fully developed flowers from each of the plants (*n* = 250 flowers). Corolla tube length (distance from the base of the corolla to the corners of the corolla mouth), corolla-entrance width, corolla-entrance height, and filament and style lengths were measured with a digital caliper (error: 0.01 mm).

A different group of flowers (*n* = 40) was collected from 10 individual plants (four flowers per plant) in order to count the number of ovules per ovary. We also assessed receptivity by submerging stigmas into a 32% hydrogen peroxide solution and using the presence of bubbling on the stigma to infer receptivity (Kearns & Inouye, 1993). Stigma receptivity of 55 flowers was recorded over 12 days after flower opening. To evaluate floral longevity, we conducted daily inspections following 235-tagged buds growing on 70 additional plants until wilting during August 2014. Fruit size measurements were taken on 40 fruits from 10 individual plants (four fruits per plant). Fruit length and width were measured with a digital caliper (error: 0.01 mm), and fruit weight with a digital scale (error 0.01 g). Seeds per fruit were also counted.

### Floral visitors

From July–September 2015, we randomly selected 58 floral patches (each composed by ∼20 plants) to determine the identity and foraging patterns of the floral visitor species. We performed 20 h focal observations (20-minute ⋅ floral patch), at different periods of the day, from 9:30–11:30 (morning), 11:50–13:50 (noon) and 14:00–16:00 h (afternoon) on different days. We used binoculars (12 × 50, Eagle Optics) to record every hymenopteran and hummingbird visit, time until some of them visited the focal patch, and the number of plants and number of flowers visited per foraging bout. We recorded the beginning of our observations as time zero and subsequent foraging events as minutes from start time. A floral visitation event was defined as the arrival of any visitor at one of the flowers of the target patch. Hummingbirds were identified with the aid of field guides (Williamson, 2001; Arizmendi & Berlanga, 2014), while insects were collected using entomological nets and sacrificed through a cyanide camera for a later identification. Total visits recordedfrom *Apis mellifera* bees and *Bombus* bumblebees were grouped and coded as visits from hymenoptera, while visits from different hummingbird species were grouped under the hummingbird category.

### Breeding system

To determine the breeding system of *P. gentianoides*, we conducted pollination experiments from July to December 2015 in a total of 50 plants with bagged buds about to open in mesh bags (1-mm bridal tulle) to exclude floral visitors. Six pollination treatments were applied to each plant: (1) autonomous-self pollination, flowers about to open were bagged to exclude floral visitors (*n* = 54 flowers); (2) hand-self pollination, flowers about to open (*n* = 54 flowers) were bagged to exclude floral visitors and after having being emasculated by cutting off the stamens, flowers were pollinated on day 8 of flower lifespan (time until stigma receptivity) by brushing their own anthers onto the stigma. After this, flowers were bagged again until fruit maturation; (3) hand-geitonogamy pollination, flowers were emasculated on their opening day and hand-pollinated (day 8) by brushing anthers from another flower of the same individual plant and pollinators excluded by enclosing the flowering branch as explained above (*n* = 65 flowers); (4) hand-outcross pollination, flowers were emasculated immediately after opening and pollinated by smearing one anther from an arbitrarily selected pollen donor onto the receptive virgin stigma, and pollinators excluded by enclosing the flower as explained (*n* = 60 flowers); (5) open-pollination, previously emasculated flowers were exposed to visitors (*n* = 49 flowers); and (6) natural open-pollination, flowers remained unbagged and exposed to natural floral visitation (*n* = 40 flowers). Fruits (capsules that change from green to tan and split open to expose seeds) from experimental flowers were collected two months later, quantified, measured (length and width) and weighed with an analytic balance (to the nearest 0.01 mg), and their seeds counted.

### Nectar production dynamics

Nectar standing crop, accumulated nectar per sexual phase and accumulated nectar throughout the lifespan of flowers were quantified to determine reward availability for pollinators. Because pollinators probably respond to nectar standing crop, we extracted the nectar available in flowers that had been exposed to floral visitors and measured its volume and concentration. Data were collected from 50 individual plants in August 2014 three times from independent flowers at 3-h intervals, at 09:00 (*n* = 93 flowers), 12:00 (*n* = 82 flowers) and at 15:00 (*n* = 82 flowers), to evaluate variation in the availability of nectar during the period of floral visitors activity. Nectar volume per flower was removed and measured by using calibrated micropipettes (5 µL) and a digital caliper (error: 0.1 mm). Sugar concentration (percentage sucrose) was measured by a hand-held pocket refractometer (range concentration 0–32° BRIX units; Atago, Tokyo, Japan), and the amount of sugar produced was expressed as milligrams of sugar after Kearns & Inouye (1993).

In a different group of 20 plants, buds about to open were randomly selected and bagged as explained above (*n* = 45 flowers) and excluded from floral visitors to let nectar accumulate. After flower opening, the accumulated nectar was extracted from the bagged flowers after the staminate phase of these flowers reached an end (first removal) and then bagged again right afterwards. Accumulated nectar was extracted from the same flowers at the end of the pistillate phase (second removal). Flowers remained bagged between nectar removals. In addition, the accumulated nectar throughout the lifespan of individual flowers from 20 individual plants was extracted one day before senescence from a different group of buds previously bagged (*n* = 40 flowers). Nectar volume and sugar concentration were measured as explained above.

### Pollinator effectiveness

From July to September 2016, we conducted a pollination experiment to compare the effectiveness of bumblebees and hummingbirds as pollinators of *P. gentianoides* flowers. Individuals (*n* = 10) of the most common visitor bumblebee species (*Bombus ephippiatus* as representative of the hymenopteran group) were captured with an entomological net, enclosed in test tubes and then used as pollen vectors. Buds ready to open from 20 plants were chosen (*n* = 40 flowers) and once they opened, flowers were emasculated and bagged with bridal netting until stigmatic receptivity. Pollination was accomplished by placing the test-tube entrance in front of the corolla of a donor flower with dehisced anthers and allowed the bumblebee to enter and leave the flower in a single event, and then repeated this procedure into the corolla of a receptive recipient flower. Bumblebees behaved normally when visiting flowers immediately after being released, without evidence of frantic flights or desperate escape attempts. We used flowers as pollen donors only from one plant to minimize possible genetic factors and to simplify our experimental design. To evaluate effectiveness of hummingbirds, a female individual of the most frequent floral visitor (*Selasphorus platycercus*) was caught with a mist net, and used as a pollen vector. Pollination was accomplished when the alive hummingbird held in hand was allowed to insert its bill once into the corolla of a donor flower with dehisced anthers and then into the corolla of a receptive recipient flower (*n* = 37 flowers from 20 individual plants), as described by Lara & Ornelas (2002); Lara & Ornelas (2003). We mimicked hummingbird behavior in probing flowers as closely as possible to avoid differences from visits in natural conditions. Pollen donors were used as explained above. After pollinations were performed the bumblebees and the hummingbird were released, and flowers remained bagged until fruit production, then fruits were weighed and the seeds per fruit produced were counted by pollination treatment as explained above.

### Data analyses

We used survival analysis (Muenchow, 1986) to analyze hymenopteran and hummingbird visitation because the observation periods were too short for all possible events to occur. For these data, the actual time of occurrence is not always known; only a minimum length of time during which the event did not occur (censored data) is always known. If a pollinator species visited a given focal plant, then it became uncensored data, and if it never occurred, then it became censored data. We used the Kaplan–Meier product-limit non-parametric method for the computation of the probability that a pollinator had not yet visited a plant 20-min after the start of the observation on each period of time and the logrank (Mantel–Cox) statistic to test differences between pollinator types.

Observed visitation frequency and number of flowers visited per foraging bout (continuous response variables) by pollinator type for each period of time (fixed effects) were analyzed using a generalized linear model (GLM) in RStudio v. 0.98.490 (R Core Development Team, 2014) with Poisson error distribution and logarithmic link function. Differences between pollinator groups per time period were evaluated with Tukey multiple comparisons (Zar, 1999).

To assess differences in fruit production among pollination treatments a GLM was performed with binomial error and a logit link function. The full GLM model included pollination treatment treated as fixed effects and fruit production as a binary response variable. Lastly, to compare the number of seed produced per fruit (continuous response variable) and pollination treatment (fixed effects) we used a GLM with Poisson error distribution and logarithmic link function. A Tukey post-hoc test was used for multiple comparisons among pairs of means of pollination treatments.

A GLM with Poisson error distribution and logarithmic link function was also used to compare nectar standing crop (volumes and amount of sugar as continuous response variables) throughout the day (fixed effects). To assess the variation in the amount of accumulated nectar throughout the lifespan of individual flowers (staminate phase, pistillate phase and accumulated throughout the life span), a GLM with Poisson error distribution and logarithmic link function was performed in R. The model incorporates flower age treated as fixed effects and the accumulated nectar (nectar volume and amount of sugar) as continuous response variables.

To evaluate differences between hymenoptera and hummingbirds in their effectiveness as pollinators, a GLM was performed with binomial error and a logit link function. The full GLM model included pollinator type treated as fixed effects and fruit production as a binary response variable. Lastly, to compare the number of seed produced per fruit (continuous response variable) by pollinator type (fixed effects) we used a GLM with Poisson error distribution and logarithmic link function. A Tukey’s post-hoc test was used for multiple comparisons among pairs of means of pollinators.

## Results

### Floral biology

In 2014, *P. gentianoides* individuals flowered from mid-July to the beginning of November, with a flowering peak occurring in September. In Table 1, we show mean values for each of the morphological attributes of *P. gentianoides*. Flowers present the three inferior petals fused, and the four stamens surrounding the pistil do not protrude from the corolla. Most flowers showed the characteristic purple color, but some flowers showed shades of color ranging from violet to blue. Flower longevity ranged from 6 to 15 d (mean ± SE, 9.9 ± 2.21 days, *n* = 235; Table 1).

**Table 1:** Flower and fruit measurements (mm) of naturally-growing *Penstemon gentianoides* (Plantaginaceae) individuals in a montane temperate forest, at the La Malinche National Park, Tlaxcala, Mexico.

	N	*n*	Mean ± SE (Min–Max)
*Flower longevity* (d)			
Lifespan	70	235	9.97 ± 2.21 (6–15)
*Flower morphology* (mm)			
Corolla length	125	250	21.06 ± 1.76 (17.69–29.24)
Corolla-entrance height	125	250	11.23 ± 2.37 (7.40–16.10)
Corolla-entrance width	125	250	9.09 ± 1.33 (5.26–13.46)
Filament length	125	250	14.69 ± 1.30 (10.40–20.25)
Style length	125	250	17.74 ± 1.85 (5.11–21.87)
Ovules/flower	10	40	218.02 ± 4.42 (131–338)
*Fruit morphology*			
Fruit weight (g)	10	40	0.11 ± 0.03 (0.04–0.18)
Fruit length (mm)	10	40	12.64 ± 1.28 (10.22–15.32)
Fruit width (mm)	10	40	6.73 ± 0.62 (5.54–8.16)
Seeds/fruit	10	40	201.48 ± 50.05 (97–353)

**Notes.**

N number of plants *n* number of flowers or number of fruits

*Penstemon gentianoides* flowers are protandrous, a floral mechanism to reduce self-pollination. Flowers on their first day of anthesis developed an eight-day male phase (staminate), followed by the ∼1–7 d female phase (pistillate phase). At the beginning of anthesis, the style is shorter than the stamens and projects far above the stamens; the stigma stands erect whereas the filaments become shorter and positioned with an ample gap between stigma and anthers (Fig. 1). Because growing stigmas make physical contact with the anthers, this very short transitional stage can be delineated as the zone at which autonomous self-pollination can theoretically take place. Stigmatic receptivity was highest between the 8–11 d.

Pollen presentation was gradual in *P. gentianoides*, restricting pollen presentation to one dehiscent anther on the first day of flower opening, and then throughout the 8 or 9 days of the staminate phase the other anthers dehisced consecutively, depending on flower longevity. At stigmatic receptivity, one of the anthers could be on its last pollen exposure event. Ovaries can form up to 218 ovules and 201 seeds per fruit on average under natural conditions (Table 1).

### Floral visitors

A total of 77 visits from four hummingbird species were registered during our observations. The most common hummingbird species was *Selasphorus platycercus* displaying territorial behavior on focal floral patches, followed by *Archilochus colubris* (66.2% and 24.6% of total visits, respectively). Other less common hummingbird visits accounted for the remaining visits (Table 2). Hummingbirds were active throughout the day. Bumblebees (*Bombus ephippiatus*, *B. huntii* and *B. weisi*) were the main floral visitors (87 visits), and their foraging activity was more restricted to warmer hours (Table 2). These hymenopteran species accounted for 95.6% of all recorded visits by insects, contrasting with the only four visits by honeybees (*Apis mellifera*).

**Table 2:** Number of visits (total number of flowers visited in parenthesis) per observational period by pollinators of *Penstemon gentianoides* flowers in a montane temperate forest, at the La Malinche National Park, Tlaxcala, Mexico. Number of visits and number of flowers visited are given for 20-min observation periods throughout the day.

Floral visitor	Morning	Noon	Afternoon	Total
*Apis mellifera*	0 (0)	3 (5)	1 (5)	4 (10)
*Bombus* spp.	13 (62)	52 (269)	22 (216)	87 (547)
*Archilochus colubris*	17 (209)	2 (14)	0 (0)	19 (223)
*Atthis heloisa*	1 (4)	1 (5)	0 (0)	2 (9)
*Hylocharis leucotis*	2 (11)	1 (1)	2 (7)	5 (19)
*Selasphorus platycercus*	10 (81)	33 (379)	8 (98)	51 (558)

**Figure 2:**
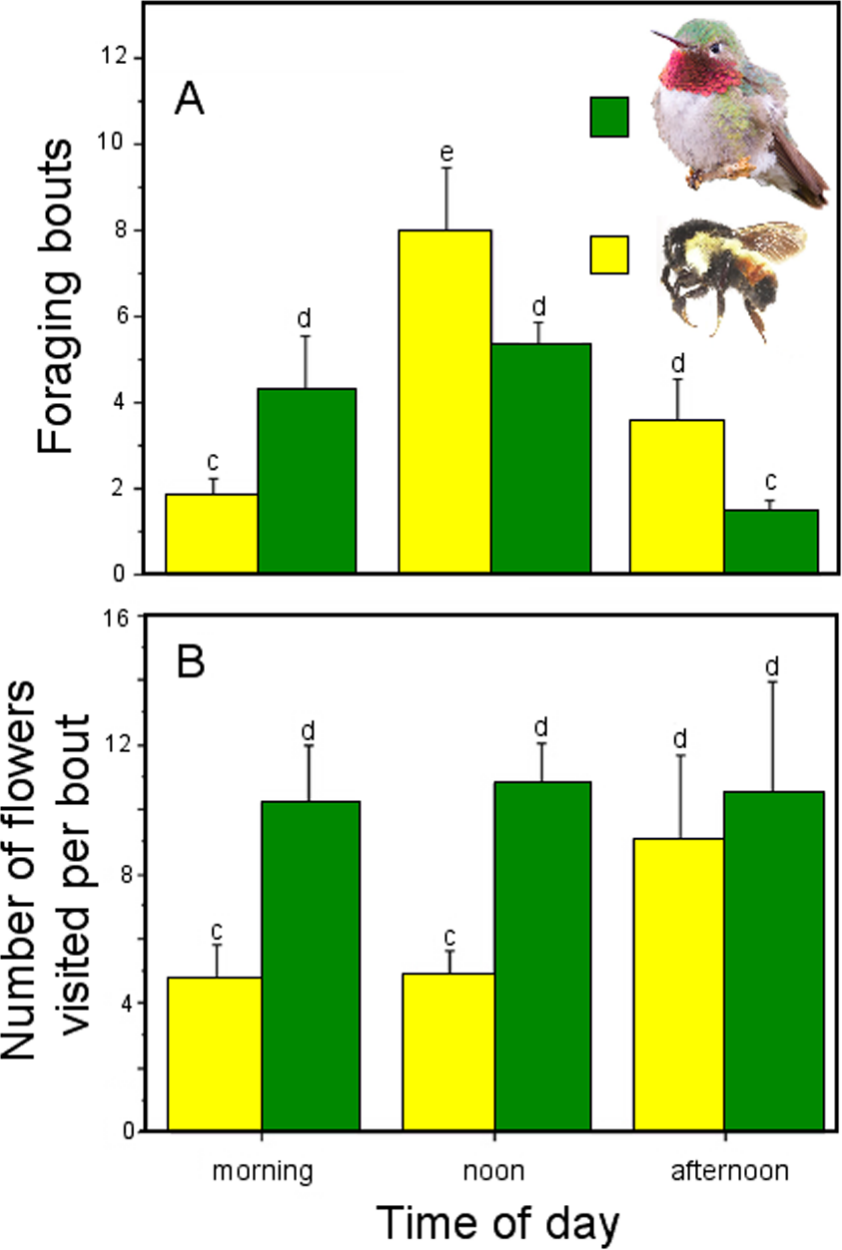
Foraging behavior throughout the day by hymenopteran and hummingbird floral visitors of *Penstemon gentianoides*. Foraging activityby hymenopteran and hummingbird floral visitors of *Penstemon gentianoides* throughout the day. (A) Number of foraging bouts. (B) Number of flowers visited per bout. Data (mean ± SE) with the same superscript letters are not significantly different between groups (*P* < 0.05).

We found no significant differences between pollinator groups (hymenopteran and hummingbirds) in the probabilities of *P. gentianoides* plants to be visited throughout the day. Waiting times for a given plant to be visited by both pollinator groups were similar at the morning (χ^2^ = 0.779, *df* = 1, *P* = 0.377), noon (χ^2^ = 1.773, *df* = 1, *P* = 0.183) and afternoon observation periods (χ^2^ = 1.422, *df* = 1, *P* = 0.233). However, we found differences in the number of foraging bouts and number of flowers visited between pollinator groups and period of time. In general, hummingbirds made more foraging bouts to *P. gentianoides* plants than hymenopteran species (GLM: pollinator group, χ^2^ = 32.678, *df* = 1, *P* < 0.0001), particularly early in the morning, but at noon and afternoon the latter exceed their visits to the plants (GLM: pollinator group ×  time period, χ^2^ = 56.328, *df* = 2, *P* < 0.0001). Interestingly, hummingbirds visited more flowers per foraging bout than insects (GLM: pollinator group, χ^2^ = 115.974, *df* = 1, *P* < 0.0001) at any period of time (GLM: pollinator group, χ^2^ = 22.363, *df* = 2, *P* < 0.0001) (Fig. 2).

### Breeding system

**Figure 3:**
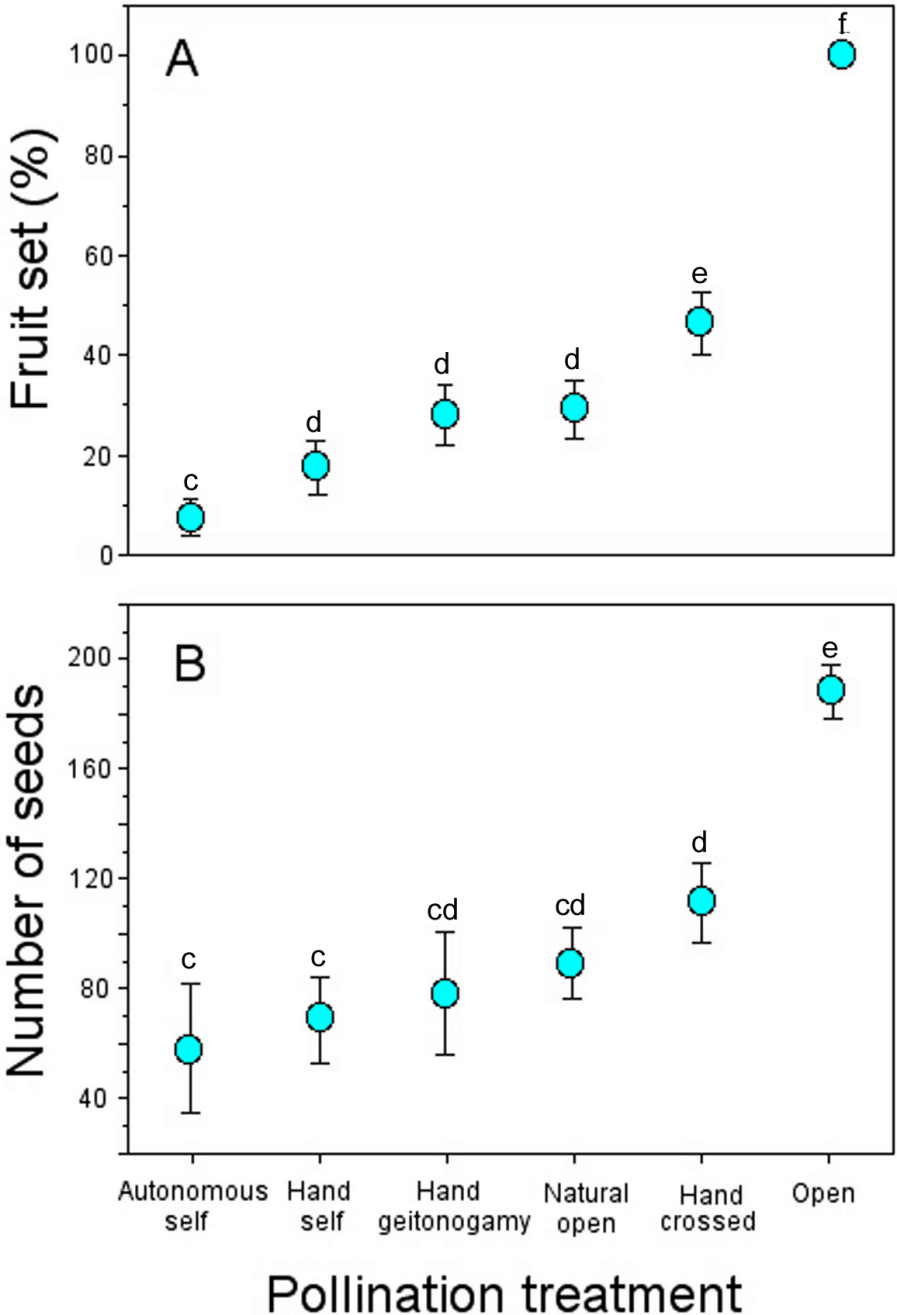
Fruit and seed production in *Penstemon gentianoides* by pollination treatment. (A) Fruit set (%, number of fruit/number of flowers) by pollination. (B) Number of seeds per fruit. Data (mean ± SE) with the same superscript letters are not significantly different between groups (*P* < 0.05).

Flowers from all pollination treatments set fruit (Table 3). However, the probability of fruit production was not independent of pollination treatment according to the GLM model (pollination treatment effects: χ^2^ = 123.09, *df* = 5, *P* < 0.0001; Fig. 3), with higher fruit production among flowers exposed to open pollination (100%), followed by hand-crossed pollinated flowers (46.7%) and geitonogamous and previously emasculated flowers exposed to natural pollination (∼25% and 28%, respectively).

**Table 3:** Number of fruits, fruit measurements, and total number of seeds of *Penstemon gentianoides* (Plantaginaceae) by pollination treatment. Data are means ± SE (Min–Max).

Treatment	*n*	Total fruits/treatment (%)	Fruit measures (mean ± SE)			Total number of seeds collected/treatment
			Weight (g)	Length (mm)	Width (mm)	
Open-pollination	40	40 (100)	0.15 ± 0.01 (0.04–0.25)	11.90 ± 0.21 (9.26–15.02)	6.45 ± 0.12 (4.84–7.89)	7,595
Natural open-pollination	49	14 (28.5)	0.06 ± 0.01 (0.02–0.16)	8.97 ± 0.46 (6.38–11.92)	5.41 ± 0.18 (4.46–7.02)	1,251
Hand-outcross pollination	60	28 (46.7)	0.08 ± 0.01 (0.00–0.23)	10.61 ± 0.50 (5.82–14.88)	5.66 ± 0.26 (4.60–8.03)	3,122
Hand-geitonogamy pollination	65	16 (24.6)	0.05 ± 0.01 (0.00–0.21)	8.97 ± 0.73 (4.96–14.58)	5.09 ± 0.33 (3.38–7.15)	1,254
Autonomous Self-pollination	54	4 (7.4)	0.02 ± 0.01 (0.00–0.04)	6.67 ± 1.77 (0.86–10.23)	4.65 ± 0.45 (4.24–5.41)	233
Hand-self pollination	54	12 (22.2)	0.05 ± 0.01 (0.00–0.13)	9.15 ± 1.01 (5.31–12.45)	4.90 ± 0.50 (3.47–6.22)	825

Number of seeds also varied significantly across pollination treatments (pollination treatment effects: χ^2^ = 123.09, *df* = 5, *P* < 0.0001). Fruits from both open pollination and hand-crossed pollination treatments were larger and produced more seeds as compared to all of the remaining pollination treatments (Table 3), and the mean differences per fruit were statistically significant (Fig. 3).

### Nectar production dynamics

**Figure 4:**
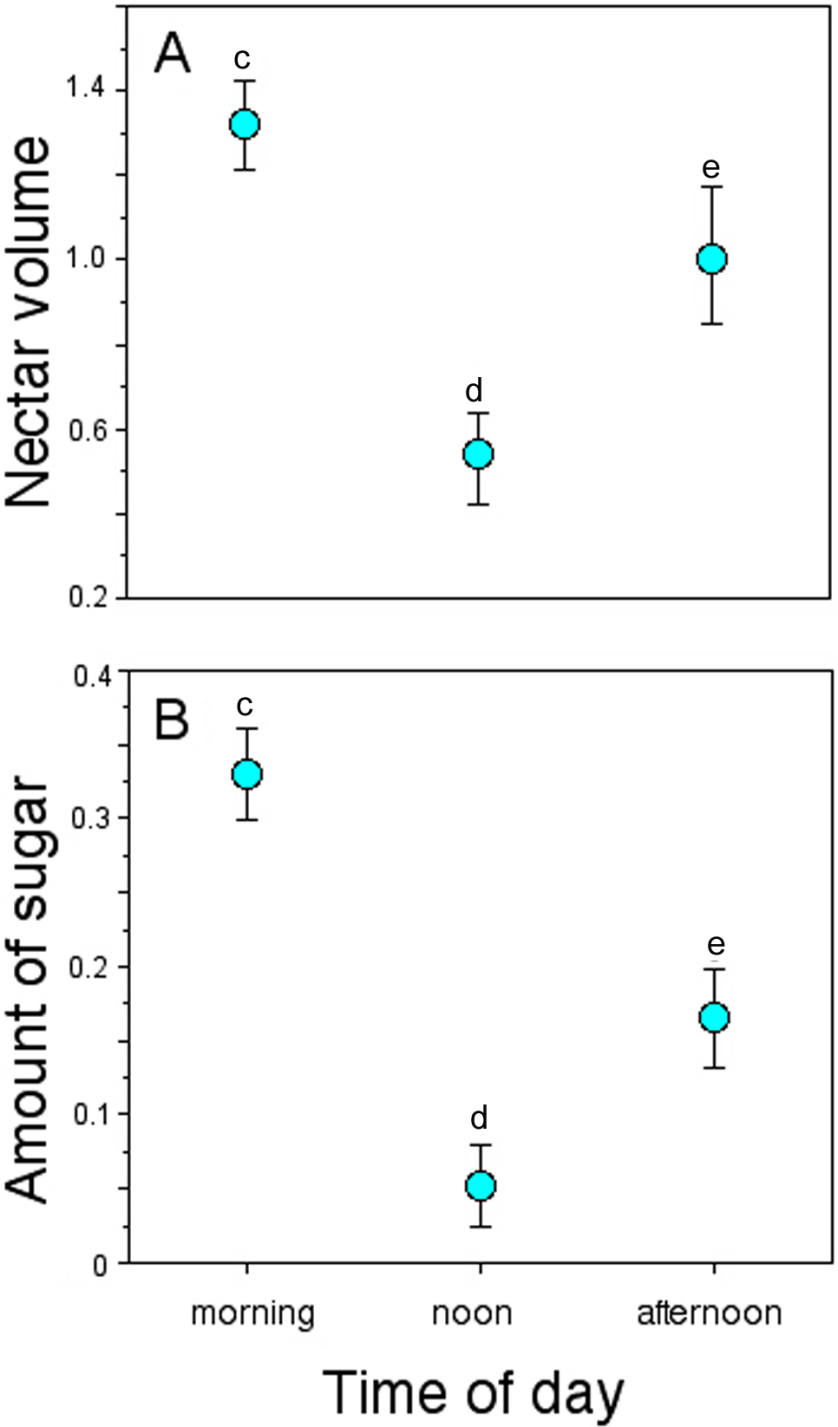
Nectar standing crop in flowers of *Penstemon gentianoides* throughout the day. (A) Nectar volume (µL per flower). (B) Amount of sugar (mg of sugar/ml per flower). Data (mean ± SE) with the same superscript letters are not significantly different between groups (*P* < 0.05).

Nectar standing crop (volume and amount of sugar) in flowers available to floral visitors varied significantly throughout the day (GLM, nectar volume: time-of-day effect, χ^2^ = 30.07, *df* = 2, *P* < 0.0001; amount of sugar: time-of-day effect, χ^2^ = 16.16, *df* = 2, *P* = 0.0003; Fig. 4). On average, flowers of *P. gentianoides* had more nectar available in the morning hours than flowers at noon or in the afternoon (Table 4).

**Table 4:** Nectar production patterns in *Penstemon gentianoides*. Nectar standing crop (volume and amount of sugar) and accumulated nectar (volume, amount of sugar, sugar concentration) in *Penstemon gentianoides* flowers.

	*n*	Mean ± SE (Min–Max)
Standing crop		
*Nectar volume (µL/flower)*		
09:00	93	3.32 ± 0.11 (0–3.95)
12:00	81	0.53 ± 0.11 (0–5.87)
15:00	81	1.00 ± 0.16 (0–4.36)
*Amount of sugar (mg/ml/flower)*		
09:00	93	0.38 ± 0.03 (0–1.05)
12:00	81	0.10 ± 0.04 (0–1.63)
15:00	81	0.21 ± 0.03 (0–0.93)
Percentage of sugar *(Brix scale)*		
09:00	93	23.3 ± 0.19 (0–25.5)
12:00	81	20.2 ± 0.04 (0–21.2)
15:00	81	21.6 ± 0.03 (0–22.3)
Accumulated		
*Nectar volume (µL/flower)*		
Staminate phase	40	12.24 ± 1.26 (0–35.71)
Pistillate phase	40	6.88 ± 0.92 (0–23.51)
Flower lifespan	40	13.57 ± 1.28 (0.23–29.98)
*Amount of sugar (mg/ml/flower)*		
Staminate phase	40	4.29 ± 0.41 (0–11.28)
Flower lifespan	40	1.88 ± 0.27 (0–6.74)
Pistillate phase	40	4.74 ± 0.47 (0.23–10.50)
*Percentage of sugar (Brix scale)*		
09:00	40	29.8 ± 0.11 (0–31.5)
12:00	40	25.2 ± 0.09 (0–27.3)
15:00	81	30.1 ± 0.16 (0–34.2)

**Figure 5:**
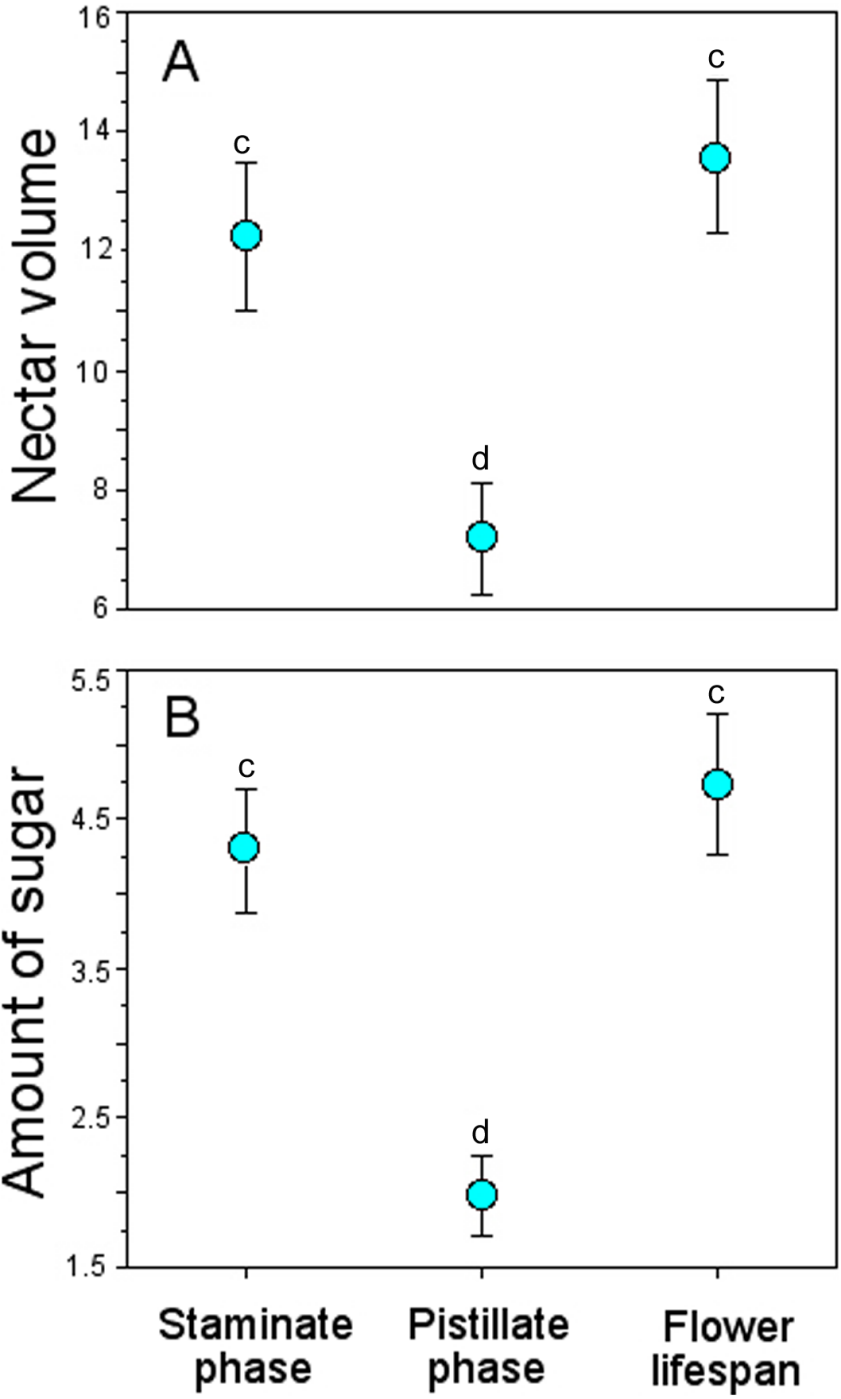
Accumulated nectar in *Penstemon gentianoides* flowers through the staminate phase, pistillate phase, and throughout the flower lifespan. (A) Nectar volume (µL per flower). (B) Amount of sugar (mg of sugar/ml per flower). Data (mean ± SE) with the same superscript letters are not significantly different between groups (*P* < 0.05).

Bagged flowers accumulated ∼13 µL and 4 mg of sugar/mL per flower throughout their lifespan, similar to those during the staminate phase, but more than twice than those in the pistillate phase (Table 4), and these differences were statistically significant (GLM, nectar volume: floral phase effect, χ^2^ = 98.94, *df* = 2, *P* < 0.0001; amount of sugar: floral phase effect, χ^2^ = 50.86, *df* = 2, *P* < 0.0001) (Fig. 5).

### Pollinator effectiveness

**Figure 6:**
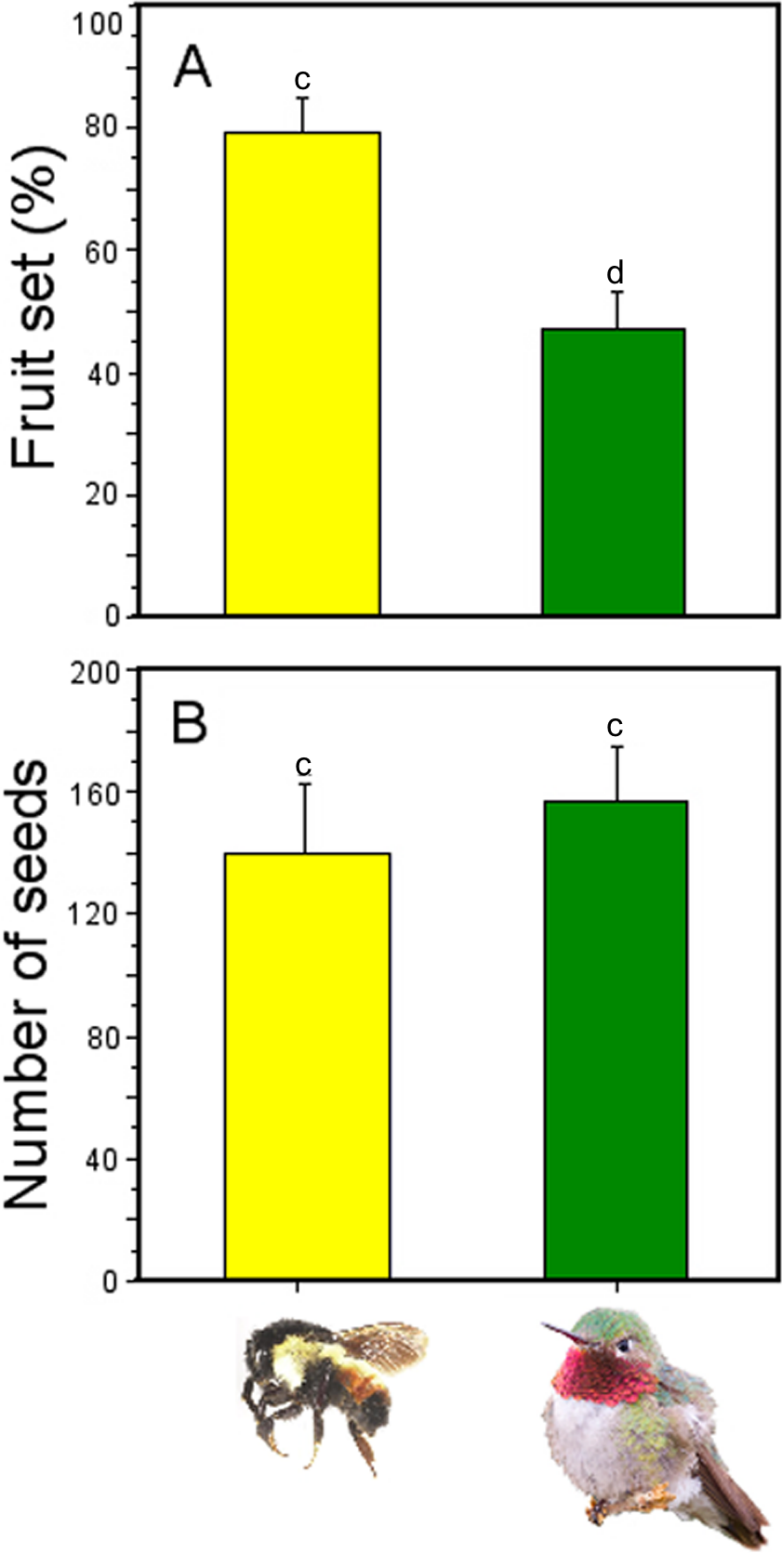
Effectiveness of bumblebees and hummingbirds as pollinators in *Penstemon gentianoides*. (A) Fruit set (%). (B) Number of seeds produced per fruit. Data (mean ± SE) with the same superscript letters are not significantly different between groups (*P* < 0.05).

Fruit production was significantly affected by pollinator type (GLM; pollinator type effects: χ^2^ = 6.82, *df* = 1, *P* = 0.008; Fig. 6), with higher number of fruits produced when flowers were pollinated by *Bombus ephippiatus* (78.7%) than flowers pollinated by *Selasphorus platycercus* (46.15%). However, there was no statistical difference in the number of seeds produced per fruit when a bumblebee (mean ± SE: 138.8  ± 23.59 seeds) or a hummingbird (156.3 ± 18.21 seeds) was the pollinator (GLM; pollinator type effects: χ^2^ = 0.34, *df* = 1, *P* = 0.558; Fig. 6).

## Discussion

We have shown that *Penstemon gentianoides* flowers fit the hymenopteran-pollination syndrome in being blue-violet, having a vestibular corolla, a lower lip in a position of a landform platform, and relatively included reproductive organs. However, flowers of *P. gentianoides* were visited and pollinated by both bumblebees and hummingbirds. Although pollination was more effective when flowers were pollinated by bumblebees, the notable high fruit production and number of seeds per fruit in flowers pollinated by hummingbirds highlights the relative importance of hummingbirds as a functional group of pollinators that might have potential evolutionary consequences to the plants.

### The hymenopteran pollination syndrome in *P. gentianoides*

The ancestral pollination syndrome in *Penstemon* is considered to be hymenoperan-adapted (Wolfe et al., 2006; Wilson et al., 2007; Wessinger et al., 2016). The vast majority of *Penstemon* species display the hymenopteran pollination syndrome, particularly species pollinated by *Osmia* and *Bombus* (Tepedino, Sipes & Griswold, 1999; Reed, 2002; Castellanos, Wilson & Thomson, 2003; Tepedino et al., 2007). However, *P. barretiae*, *P. centranthifolius*, *P. newberryi*, *P. rupicola*, *P*. *pinifolius*, *P*. *ramosus*, and *P*. *superbus* to name some species*,* display floral traits linked to hummingbird pollination, such as long and narrow corollas, colored in shades from purple pink to red (Bateman, 1980; Lodewick & Lodewick, 1992; Lange & Scott, 1999; Tepedino, Sipes & Griswold, 1999; Datwyler & Wolfe, 2004; Castellanos et al., 2006; Walker-Larsen & Harder, 2001; Broderick, 2010). Similarly, several species that present floral traits initially linked to insect pollination, such as *P. pseudospectabilis* and *P. bridgesii,* increase their seed production when visited by hummingbirds, suggesting mixed pollination systems (Carpenter, 1983; Lange & Scott, 1999). Here we showed that *P. gentianoides* is effectively pollinated by both hymenoptera and hummingbirds, even while maintaining its auto-compatibility capacity; a phenomenon previously described in numerous species such as *P*. *centrantifolius*, *P*. *rostrifolius*, *P*. *ellipticus*, *P*. *palmeri*, *P*. *penlandii* and *P*. *pseudospectabilis* (Lange & Scott, 1999; Tepedino, Sipes & Griswold, 1999; Lange, Scobell & Scott, 2000; Walker-Larsen & Harder, 2001; Tepedino et al., 2007).

Some studies have documented variation in several *Penstemon* floral traits, particularly flower size, corolla shape and color, in addition to the offered nectar rewards (Keck, 1937; Straw, 1956; Freeman et al., 1984). However, as Lange & Scott (1999) stated, variation in the amount of nectar rewards as a characteristic to be linked to a given pollination syndrome has been overlooked. In our study, the intensity of pollinator visitation through the day was differential, displaying a pattern linked to nectar availability. For instance, the amount of nectar available in *P*. *gentianoides* flowers in the morning hours (volume: 1.32 µL; amount of sugar: 0.38 mg/mL) is about twice the amount collected at noon (0.53 µL; 0.10 mg/mL) and afternoon (1.00 µL; 0.21 mg/mL). Coincidentally, *P. gentianoides* flowers were more visited by hummingbirds early in the morning, when more and dilute nectar is available. Water input in semi-concentrated nectar volumes is a hummingbird preference already documented (Baker, 1975; Pyke & Waser, 1981). However, at noon, when flowers had less nectar but sugar concentration was higher, bumblebees were the ones who visited the most. Interestingly, at this particular schedule is when hummingbirds substitute the lack of high nectar volumes by probing more flowers per foraging bout.

On average, we found that the accumulated nectar during the male phase (staminate phase) of *P. gentianoides* flowers was higher than the amount accumulated during the female phase (pistillate phase). The variation in the capacity to produce and replenish nectar throughout the sexual phases of *Penstemon* has been formerly documented. For example, Castellanos, Wilson & Thomson (2002) compared nectar replenishment patterns in protandrous flowers of *Penstemon* manly visited by several species of Hymenoptera (*P. speciosus*), bumblebees (*P. strictus*) or by hummingbirds (*P. barbatus*). Species with the hymenopteran pollination syndrome quickly replenished a small amount of concentrated nectar, and hummingbird-adapted species refilled their nectaries to a higher level with more dilute nectar. In these *Penstemon* species, male- and female-phase flowers are intermingled within a plant, so nectar in a female-phase flower may actually be serving male function in an adjacent flower even after its own stigma has been saturated. Therefore, there is little reason to expect an association between nectar production and sexual phase (Castellanos, Wilson & Thomson, 2002). In *P. gentianoides*, we found that the amount of dilute nectar accumulated during the extended 8-d male phase was significantly higher than the amount accumulated during the 1–7-d female phase, and stigmatic receptivity was highest between the 8–11 d. Secreting large quantities of dilute nectar during the staminate phase may encourage non-territorial hummingbird pollinators to revisit plants while keeping low the rate of geitonogamy. Another reason for producing more dilute nectar during the staminate phase and for reducing the amount and the extent of the pistillate phase is conservation of energy and water at high elevations, particularly when nectar production and replenishment and pollination intensity jointly affect seed production (Ornelas & Lara, 2009).

Lara & Ornelas (2008) found in *P. roseus* a daily secretion rate of 0.3 mg of sugar per flower per day, a relatively low amount of sugar relative to nectar sugar production in hummingbird-adapted *Penstemon* species (range 1.5–5 mg sugar per flower per day; Lara & Ornelas, 2008 and references therein). To our knowledge, there is no comparative data for bee-adapted *Penstemon* flowers except that they produce more concentrated nectar (e.g., Freeman & Worthington, 1985; Kimball, 2008; Kimball & Campbell, 2009). However, Lara & Ornelas (2008) hypothesized that the daily secretion rate in *P. roseus* was intermediate between hummingbird- and bee adapted *Penstemon* species. They further suggested that the nectar secretion patterns (large volumes of dilute nectar) in the usually bright red flowers of *P. roseus* represents a shift toward hummingbird pollinations, in which a ‘despecialized’ *Penstemon* species attracts high-energy pollinators (hummingbirds) and profits from outcrossing, but retains bee-syndrome floral traits and low sugar production. Further comparative studies are required to test these ideas.

### Ancestral pollination syndrome and secondary functional groups

Evolutionary shifts from insect pollination to more efficient hummingbird pollination have occurred repeatedly in *Penstemon*, with minimally ten and up to 21 origins of hummingbird pollination (Wolfe et al., 2006; Wilson et al., 2007; Wessinger et al., 2016). The ancestral hymenopteran pollination toward more derived hummingbird pollination has resulted in the convergent evolution of floral traits commonly present in hummingbird-adapted *Penstemon* species (Wilson et al., 2004; Wilson et al., 2007). However, the floral phenotypes consonant with hymenopteran and hummingbird pollination syndromes are not always predominantly adapted to their predicted principal pollinators and flowers may have suites of mixed floral traits and a mixed set of floral visitors. For instance, the floral morphology of *P. roseus* corresponds to the *Penstemon* bee-pollination syndrome that has undergone ‘despecialization’ in the sense of the flowers taking hummingbirds as pollinators while still having the characters that allow locally rare bumblebee pollination (Wilson et al., 2006; Lara & Ornelas, 2008). Species of *Penstemon* that have acquired hummingbirds as pollinators are pink or magenta. This shift in color seems to have occurred in nearly all species of *Penstemon* that have begun a shift toward hummingbird utilization (Wilson et al., 2006), including the ‘despecialized’ *P. roseus* (Lara & Ornelas, 2008). In contrast, the morphology of the violet *P. gentianoides* flowers represents an intermediate stage regarding shifts from bumblebee pollination toward hummingbird pollination because both groups of floral visitors are effective pollinators.

Our data showed that *P. gentianoides* is self-compatible. However, cross-pollinated (xenogamous) flowers produced more fruits and seeds than self-pollinated flowers, highlighting the importance of pollen flow mediated by floral visitors for the successful seed production in this plant species. Although we only measured female reproductive success, previous studies have shown than male success in this genus could have important implications. For example, reproductive success in *Penstemon* species through male function depends heavily on the packaging and gradual presentation of pollen to pollinators (Thomson et al., 2000; Castellanos, Wilson & Thomson, 2003; Castellanos et al., 2006). In this regard, flowers of *P. gentianoides* opened the anthers and released pollen gradually, a response that might be related to the high rates of flower visitation by bumblebees (see also Williams & Thomson, 1998). Accordingly, the number of ovules per ovary and the number of seeds produced by *P. gentianoides* flowers exposed to open pollination suggest that pollen is not limited, a feature usually linked to pollinator efficiency (Barrett, 2014). However, in our pollination experiments crossed pollinated flowers produced fewer seeds than those exposed to open pollination. This discordance might suggest that this plant species requires greater amounts of pollen (pollination intensity) and a scheduling of pollen presentation for a greater pollination success (Thomson et al., 2000; Ornelas & Lara, 2009), which could not be matched by our hand-pollination treatment. These aspects of pollen receipt should be considered in future pollination experiments on this plant species.

Evolutionary shifts between pollination syndromes presumably take many generations of incremental change in the many characters involved (Thomson et al., 2000). However, the failure to resolve relationships at the species level within the crown group using fast-evolving markers (Wolfe et al., 2006), gene tree discordance due to incomplete lineage sorting, introgression or hybridization (Wolfe, Xiang & Kephart, 1998; Datwyler & Wolfe, 2004; Wolfe et al., 2006), and the difficulty to confidently infer short internal branches, even using RADseq phylogenomic analyses, all support the hypothesis that *Penstemon* has experienced a recent and rapid radiation (Wessinger, Hileman & Rausher, 2014; Wessinger et al., 2016). This suggests that major pollinator shifts, from hymenopteran- to hummingbird-adapted pollination between closely related species, could occur rapidly in a few scores of generations in which intermediate stages are ephemeral (see also Thomson et al., 2000). Accordingly, the higher frequency of hummingbird floral visits and their effectiveness as pollinators is a relatively recent phenomenon, in which the more reliable hummingbirds are allowed to take over the ancestral hymenopteran pollination system by simply regulating the amount of nectar present in the flower.

By means of phylogenetic meta-analysis, (Rosas-Guerrero et al., 2014) assessed whether floral traits predict the most effective pollinators of plants and whether the predictability of pollination syndromes was associated with the pollinator functional group. In general, their findings supported the Stebbins’ principle (Stebbins, 1970) of convergent evolution of floral traits driven mainly by the most effective pollination functional group. When syndromes failed, they found in the pollination networks that the pollinator predicted by the syndrome was still present within the pollinator assemblage. In these cases, the most effective pollinator was often the main secondary pollinator of the syndrome and may represent the ancestral pollination system of the plant lineage (Rosas-Guerrero et al., 2014). According to this, transitions from the ancestral hymenopteran pollination syndrome to more derived hummingbird pollination syndrome in *P. gentianoides* may be initiated when the quantity or quality of visits by hummingbirds is increased compared to the less efficient ancestral pollinator group.

Along the distributional range of *P. gentianoides* in Mexico, its primary habitat is related to montane environments, particularly pine and fir forests, mountain hayfields and grasslands (Calderón de Rzedowski & Rzedowski, 2002). Coincidentally, these habitats are also reservoir for species-rich hummingbird communities composed of both resident and migratory species. For that reason, although native *Bombus* species registered throughout our study are still currently important for *P. gentianoides* pollination, contemporary changes in the pollination environments (e.g., *Bombus* pollinators experiencing population declines; Duennes et al., 2012; Duennes & Vandame, 2015; Hatfield et al., 2015; Vandame & Martínez López, 2016) could change depending on the composition of pollinator assemblages and consequently make the described mixed system to increase the potential for a transition from the ancestral pollinator syndrome to hummingbird pollination. When hummingbird visitation is sufficiently reliable, we would expect *P. gentianoides* flowers to experience selection to attract, reward or increase the efficiency of hummingbirds but also for those that deter or decrease removal by bumblebees (Castellanos, Wilson & Thomson, 2004; Zung et al., 2015). Further manipulation of traits linked to the attractiveness of *P. gentianoides* and the efficiency of its pollinators would be needed to test these ideas.

In short, we have shown that *P. gentianoides* has a mixed pollination system. Although pollination efficiency is higher among flowers visited by hymenoptera, the noteworthy percentage of fruit production and number of seeds per fruit derived from hummingbird pollination supports the importance of hummingbirds as a functional group. To determine whether *P. gentianoides* is transitioning from insect to bird pollination or whether its mixed pollination system represents a stable and very effective reproductive strategy additional studies are required.

## Additional Information and Declarations

### Field Study Permissions

The following information was supplied relating to field study approvals (i.e., approving body and any reference numbers): The field research reported here was performed using the required permit (SEMARNAT No. FAUT-0296).

### Data Availability

The following information was supplied regarding data availability: Our raw data was only used to obtain floral traits values, foraging waiting times of pollinators and statistics, and are included in Tables 1– 4.
